# Investigating Context in How Clinicians Make Sense of AI: Qualitative Interview Study

**DOI:** 10.2196/99117

**Published:** 2026-11-13

**Authors:** Simone Mingels, Hannah Piehl, Rianne Fijten, Ricky Janssen, Bart Penders

**Affiliations:** 1 Department of Radiation Oncology, GROW Research Institute for Oncology and Reproduction, Maastricht University Medical Centre+, Paul-Henri Spaaklaan 1, Maastricht, Limburg, 6229 GS, The Netherlands, +31 0433881863; 2 Maastro, Maastricht Radiation Oncology Institute, Maastricht, The Netherlands; 3 Department of Health, Ethics & Society, Care and Public Health Research Institute (CAPHRI), Maastricht University, Maastricht, The Netherlands; 4 Department of Health, Ethics & Society, Mental Health and Neuroscience Research Institute (MHeNs), Maastricht University, Maastricht, The Netherlands

**Keywords:** AI, decision support, science and technology studies, human-computer interaction, sociotechnical systems, adoption

## Abstract

**Background:**

AI-driven clinical decision support systems (AI-CDSS) are increasingly being introduced to enhance clinical decision-making, yet their adoption in health care remains limited. Research has examined technical, organizational, and contextual barriers to adoption, but less attention has been paid to how knowledge, values, and responsibility around AI-CDSS are constructed and negotiated in practice.

**Objective:**

This study examines how clinicians and AI experts make sense of AI-CDSS by reanalyzing qualitative interview data through a feminist and constructivist science and technology studies (STS) perspective. Whereas the original study examined informational needs, this reanalysis asks how participants construct relevant knowledge, evaluate the value of AI-CDSS, and negotiate appropriate use and responsibility in clinical practice.

**Methods:**

The authors conducted a reanalysis of semistructured interviews held in 2024 with clinicians (n=8) and AI experts (n=8) in the Netherlands. The original study focused on informational needs for AI-CDSS. For this analysis, a deductive coding framework based on European Commission guidelines for trustworthy AI was first applied, and the material was then inductively reorganized into 5 empirical themes. Subsequently, feminist and constructivist STS concepts were used as sensitizing concepts in an abductive analysis, and these concepts were brought into dialogue with the empirical themes to examine the sociotechnical relations underlying participants’ accounts.

**Results:**

*Situated knowledge* shows that the informational needs of clinicians are not universal but relate to their roles and specific clinical contexts, with understanding oriented less toward technical transparency than toward actionable guidance, relevance, and integration into decision-making. *Standpoint theory* illuminates differences between stakeholder priorities: clinicians emphasize information on how to use and act on AI outputs within existing workflows, whereas AI experts focus more on providing technical characteristics and performance metrics. *Tinkering* captures how clinicians experiment with AI systems in practice to develop situated, lay forms of expertise. *Situated value* highlights that perceptions of an AI-CDSS’s worth are relational, with clinicians valuing novel, context-sensitive insights and developers prioritizing performance metrics. Finally, *response-ability* reveals how trust and accountability are largely delegated to institutional structures, while clinicians report limited legal, organizational, and structural support to respond meaningfully to uncertainty or errors when using AI-CDSS.

**Conclusions:**

Reanalyzing the same interview material through an abductive STS approach makes sociotechnical dimensions visible that were not the focus of the original analysis. Rather than treating informational requirements as universal or adoption as primarily a matter of technical performance and communication, the findings show how knowledge, value, and responsibility are constituted through situated professional practices and institutional arrangements. Processes and considerations regarding how clinicians make sense of AI-CDSS are not universal but are shaped by social, cultural, and institutional contexts. This analytical perspective underscores the need for context-sensitive, multistakeholder strategies to support meaningful and responsible AI adoption in clinical practice.

## Introduction

AI is becoming an integral part of health care, with the potential to revolutionize clinical decision-making. By predicting outcomes and offering treatment options tailored to individual patients, AI-driven clinical decision support systems (AI-CDSS) promise a more personalized and efficient approach [1-3]. However, despite its potential, the adoption of AI-CDSS in clinical practice remains slow [4].

This slow or lack of adoption has been studied thoroughly by examining factors such as technical performance, data quality, workflow integration, and regulatory compliance [5]. These studies have highlighted that AI can be difficult for clinicians to interpret and explain, and that its integration often demands significant adjustments to established clinical routines [6].

Considerations about algorithmic bias, legal liability, and ethical concerns, as well as uncertainty about the effects of these systems regarding roles and responsibilities, further complicate the adoption of AI in health care [7-9]. While these studies provide valuable insights into social, organizational, and contextual factors, alongside technical characteristics, they often tend to conceptualize these dimensions as determinants, barriers, or facilitators of adoption. This overlooks the ways in which trust, professional identity, and medical practices shape how AI is understood and used, as well as processes through which stakeholders negotiate what constitutes useful, trustworthy, and appropriate AI in clinical practice. Addressing these dimensions requires approaches that explore not only the functionality of AI systems but also how they fit into the social and institutional environments of health care to contribute to meaningful forms of adoption.

Human-computer interaction (HCI) and technology adoption research are among the fields that have increasingly sought to address AI as a sociotechnical system. A sociotechnical perspective holds that social and technological dimensions are inseparable and must be examined in their mutual constitution rather than in isolation [10]. However, despite this shift toward relating social and technical factors to each other, HCI operationalizes social context through structured frameworks, such as the technology acceptance model [11] and the unified theory of acceptance and use of technology [12], as well as determinants intended to support implementation or adoption. As a result, much of the literature on clinical decision support systems (CDSS) continues to rely on structured frameworks or models to incorporate social and contextual factors [13-15]. In this paper, we turn to perspectives from other disciplines that foreground questions of power, governance, and contestation in the development and use of AI-based systems in health care—questions that are less often the primary object of technology adoption–oriented research [16,17].

Science and technology studies (STS) provides a distinct perspective by examining the relationship between science, technology, and the societal structures within which they are developed and deployed. STS is an interdisciplinary research field exploring how scientific knowledge and technological advancements are shaped by, and in turn shape, cultural, political, and economic contexts [18]. Closely related to critical branches of the public understanding of science (PUS), STS highlights that science and technology are intertwined with historical, societal, and cultural dimensions and cannot be viewed separately from the sociotechnical surroundings within which they are constructed. Moreover, STS centers on how “scientific and technological configurations of illness and treatment are themselves charged with social meaning and importance” (p. 469) [19]. Therefore, understanding AI adoption involves not only identifying social, technical, or organizational factors that influence implementation but also revolves around questions such as: Who decides what counts as *working well* for an AI-CDSS? and What does *adoption* or *correct use* mean?

These questions should not be confined to social scientists or STS researchers but should also be taken up by stakeholders directly involved in the development and implementation of AI-CDSS as a prerequisite for responsible, equitable, and reflective deployment. Accordingly, Wagman and Parks [16] call for closer engagement between STS and HCI, which they argue can offer “ways of thinking about the complexities of the social, user identities, and power dynamics that can enrich the process of conceptualizing, designing, and developing social machines” (p. 4).

A substantial body of scholarship in STS and the sociology of health has examined digital health technologies by focusing on how they reconfigure relationships, responsibilities, and practices within health care settings [17,20-22]. We aim to extend these approaches to AI systems, whose opacity, scalability, and perceived autonomy introduce distinct challenges that warrant renewed examination of clinical responsibility and trust [23,24].

In our previous study, clinicians were interviewed about their informational needs when using AI-CDSS [25]. An underlying assumption in that study was that providing clinicians with more and *better* information would raise awareness and ensure safe and appropriate use of AI-CDSS. This reflects what STS and PUS scholars refer to as the *deficit model*, which assumes that hesitancy or mistrust toward an emerging technology is the result of a lack (ie, a deficit) of knowledge [26]. However, this perspective oversimplifies the dynamics at play. Trust in AI is not built solely by closing a knowledge gap but is also shaped by factors such as perceived relevance, institutional support, and the alignment of technology with existing practices and values [27-29]. Recognizing the limitations of the deficit model underscores the need for approaches that examine how information is constructed, translated, challenged, and negotiated in specific contexts.

Through an STS lens, this study reanalyzed interview material to examine how clinicians and AI experts construct requirements and concerns regarding AI-CDSS in clinical practice. Rather than aiming to deliver concrete implementation strategies, design guidelines, or policy recommendations, our objective was to unpack the sociotechnical conditions that shape perceptions of knowledge, value, and responsibility. In this process, STS concepts provided conceptual resources for interpreting patterns in the data without dictating our empirical findings.

## Methods

### Study Design

This qualitative study reanalyzes interview data to demonstrate how empirical material collected under a different analytical orientation can reveal additional sociotechnical dimensions. The aim of the original research was to explore the informational needs of clinicians regarding the use of AI-driven recommendations in medical decision-making [25]. The data were originally gathered from two perspectives: (1) user-centered preferences of clinicians and (2) technical and safety requirements identified by AI experts. AI experts were determined as individuals with direct experience in developing and/or implementing AI systems for use in health care. The selection of the 2 groups in the original research was based on the assumption that the informational needs may go beyond the expressed preferences of clinicians and require expert-identified information for the informed and safe use of AI in clinical settings. This paper was written in accordance with the Consolidated Criteria for Reporting Qualitative Research (COREQ) checklist (Checklist 1) [30].

### Research Group

SM is a PhD student in Clinical Data Science (CDS) at Maastricht University. Her research focuses on how to communicate information about AI to health care professionals and patients, with the aim of supporting understanding of AI-based tools. HP is a PhD student at CDS and is affiliated with the Department of Health, Ethics and Society (HES). Her research explores the trustworthiness of AI in health care, focusing on how negotiations around and disruptions of trust shape the implementation of AI. RF is an assistant professor at CDS. Her research explores the application of AI in the health care domain, with an emphasis on the various aspects that influence the clinical adoption of AI, such as trust, informational needs, effectiveness, and more. RJ is an assistant professor at HES. Her research explores how new digital technologies and medical diagnostics contribute to care practices and the changing roles of, and relationships between, patients, health care providers, and health services more broadly. BP is an associate professor of Biomedicine and Society at HES. His research examines how credibility, integrity, and collaboration shape trustworthy knowledge, and how scientific norms evolve under institutional and technological change.

### Reflexivity and Research Process

This reanalysis was initiated after SM and HP collaborated on the original study [25]. Throughout the initial analysis phase, the research team (SM and HP) observed significant thematic depth in the data. While not relevant to the original research question, it warranted a dedicated investigation from a distinct theoretical perspective.

To explore dimensions that were intentionally excluded from the original research scope, we investigate how clinicians’ understanding of AI-CDSS extends beyond the information requirements constructed by clinicians and AI experts. We initially focused on themes of trust in AI, which is the main area of expertise of one researcher (HP). However, during the reanalysis process, we extended the analytical focus beyond trust by drawing on STS concepts to take broader sociotechnical perspectives into account—which affect, but also go beyond, trust in technology.

While one set of researchers (HP, RJ, and BP) had previous experience applying STS concepts to empirical material, the remainder (SM and RF) were unfamiliar with STS. Furthermore, over half of our team works in a department that has a generally optimistic and positivist stance toward the dissemination and implementation of AI, which might have shaped the research process and the reanalysis of the data.

### Study Participants

The original study used a combination of convenience and snowball sampling to recruit participants between July and November 2024. Potential participants were identified through the research team’s professional networks and targeted LinkedIn (Microsoft Corp) searches, with invitations and study information folders distributed via email or personal messages. Due to interview participants’ time constraints, 8 individuals per group were set as the target for recruitment rather than basing recruitment on theoretical saturation. The original study established inclusion criteria requiring clinicians to be active practitioners in the Netherlands, while excluding any potential participants with less than 2 years of relevant work experience. Clinicians were not required to have any prior knowledge of AI systems. AI experts were included based on direct professional involvement in the development, evaluation, or implementation of AI systems in university and/or health care settings. All participants were based in tertiary care settings, either academic medical centers or specialized institutes, where clinicians practiced and AI experts developed, implemented, or evaluated AI systems. An extensive overview of the participants is provided in Table 1.

**Table 1.** Recruitment and participation information, demographic and professional characteristics, and interview settings of the 16 participants interviewed for the original study between August and November 2024 in the Netherlands.

Characteristics	Clinicians (n=8)	AI experts (n=8)
Recruitment and participation		
Total participating/total invited, n/N (%)	8/26 (31)	8/11 (73)
Declined—lack of time, n	8	0
Declined—no reason provided, n	10	3
Demographics		
Gender, n		
Men	6	5
Women	2	3
Median age (y), (IQR)	44 (29-59)	37 (28-48)
Professional roles		
Median work experience (y), (IQR)	14 (2-22)	11 (3-21)
Specialties/roles, n		
Internal medicine	2	—^a^
Oncology	2	—
Gastroenterology	2	—
Cardiology	1	—
Radiology	1	—
Medical imaging AI	—	3
Innovation and implementation management	—	3
Data infrastructure and FAIR^b^ principles	—	2
Previous professional AI experience, n		N/A^c^
Prediction models	5	
Imaging	2	
Generative AI	1	
Interview setting		
Interview language, n		
Dutch	8	6
English	0	2
Format, n		
Online	4	6
Face-to-face	4	2

a Not applicable.

b FAIR: findability, accessibility, interoperability, and reusability.

c N/A: not available (subject matter experts).

### Interview Data

SM conducted semistructured interviews with 8 clinicians and 8 AI experts between August and November 2024. Interviews were held either online through Microsoft Teams or face-to-face at the hospital or university and lasted between 30 and 60 minutes. While most sessions were conducted in Dutch, 2 interviews with AI experts were held in English. The interviews were transcribed verbatim, and all Dutch interviews were translated into English. No field notes were made during the interviews.

The interview guide contained questions eliciting participants’ reflections on their experiences with AI applications in health care settings and their involvement in the development of AI systems for clinical use (Textbox 1). Clinicians were asked what information they would ideally want to receive, while AI experts were asked what information they believe should be communicated to end users. In this context, clinicians are regarded as the end users of the AI-CDSS. In the final segment of the interview, all participants were presented with 3 examples of documentation tools—Model Card [31], Model Facts [32], and the TRIPOD-AI (Transparent Reporting of a Multivariable Prediction Model for Individual Prognosis or Diagnosis—Artificial Intelligence) checklist [33,34]—which served as prompts for discussion about clarity, usefulness, and relevance in supporting transparency in AI-CDSS. These materials functioned as discussion prompts to explore participants’ perceptions of possible transparency formats but were not themselves the focus of the analysis. The interview guide and examples (Multimedia Appendices 1 and 2) were based on a Bayesian Network from a public repository to reflect moderate model complexity, with full construction details described in Mingels et al [25]. No repeat interviews were conducted.

Textbox 1. **Outline of the interview guide topics for clinicians and AI experts used in the original research [25].**

**Clinicians**

- Demographic information (age, gender, job description, and work experience)
- Experience with AI in care settings
- Currently received information about AI models
- Information they would prefer to receive
- Examples of the Model Card, TRIPOD-AI (Transparent Reporting of a Multivariable Prediction Model for Individual Prognosis or Diagnosis—Artificial Intelligence) checklist, and Model Facts were shown, and participants were requested to provide feedback on the amount, structure, and comprehensiveness of the information

**AI experts**

- Demographic information (age, gender, job description, and work experience)
- Experience developing AI for care settings
- Provided information concerning AI models
- Information they think they should provide
- Examples of the Model Card, TRIPOD-AI (Transparent Reporting of a Multivariable Prediction Model for Individual Prognosis or Diagnosis—Artificial Intelligence) checklist, and Model Facts were shown, and participants were requested to provide feedback on the amount, structure, and comprehensiveness of the information

While we recognize that the original interview guide and interviews were designed from a post-positivist perspective, focusing on practical requirements and outcomes, the richness of the qualitative data nevertheless provided insights into how participants engaged in making sense of AI-CDSS in clinical practice. We acknowledge that the original research design constrained the extent of theoretical engagement and that the interview guide did not explicitly explore the dimensions we discuss in this publication. However, the depth and variability of responses allowed us to identify themes relevant to social, institutional, and moral dimensions of AI adoption, which go beyond the original focus on informational needs. This illustrates that even studies oriented toward practical outcomes inevitably capture the complexities of health care AI, highlighting the value of reinterpreting data through a theoretical lens different from that originally intended.

### Data Analysis

This interpretive study combines deductive and inductive coding strategies. Initially, deductive codes based on the European Commission’s guidelines for trustworthy AI [35] were collaboratively applied by SM and HP to all interview transcripts using ATLAS.ti Web (version 25.0.1; ATLAS.ti Scientific Software Development GmbH). This provided insights into how participants linked specific informational needs to the European Commission’s conceptualization of trustworthiness, as notions of trust appeared both organically and implicitly throughout the interview data.

Following this, SM and HP collaboratively reviewed and restructured the coded material, carrying out an inductive thematic analysis [36]. Several thematic areas were created from this reorganization of the data. The themes were information overload vs information deficit, trust through experimentation, understanding the output, institutional trust and responsibility, and clinical relevance.

During the reorganization, one researcher (HP) identified close affinities with feminist and constructivist STS concepts. Constructivist concepts assume that scientific knowledge and technological design are socially and materially constructed, and are therefore a product of their surrounding social and material processes. Feminist STS critically examines power relations that shape knowledge production and the development of technology, questioning whose perspectives are included and what assumptions guide them.

Following extensive discussions with the supervisory team familiar with STS (BP and RJ), SM and HP linked the themes to STS concepts to provide analytic structure and to make them accessible to audiences less familiar with feminist and constructivist STS. Specifically, situated knowledge was used to examine how knowledge claims depend on social and professional position; standpoint theory to examine differences in perspectives and epistemic priorities across stakeholder groups; tinkering to examine experimentation and practical knowledge-making in use; situated value to examine how the worth of AI-CDSS is constituted in relation to practices and priorities; and response-ability to examine how responsibility is distributed and enacted within institutional arrangements. These concepts were introduced after inductive thematic reorganization and were used abductively to interpret and reconsider empirical patterns. Their relation to the interview material is described in the “Results” section.

In summary, our analytical process followed a 3-step sequence: initial deductive coding mapped data to the European Commission’s trustworthy AI framework; inductive thematic reorganization then clustered these codes into core empirical themes; and finally, abductive theorization, following principles of abductive reasoning, integrated prior theoretical knowledge of STS concepts to make sense of the practical complexities and tensions encountered in the empirical material. The 2 frameworks used in the analysis served different epistemic and analytical functions. The European Commission’s framework for trustworthy AI was used deductively as an initial normative coding framework, providing predefined dimensions through which participants’ accounts could be systematically examined. This approach does not share the epistemic commitments of feminist and constructivist STS, which instead informed the subsequent interpretive phase. Abductive thematic analysis is distinct from both inductive and deductive approaches. It specifically addresses scenarios where empirical observations are brought into iterative dialogue with theoretical literature to interpret complex phenomena [37,38]. In this study, we viewed these inductively created themes through STS sensitizing concepts to unpack the underlying sociotechnical dynamics. The analysis consequently treats the 2 frameworks as sequential rather than epistemologically integrated: the first structured the initial empirical analysis, while the second enabled a critical reinterpretation of what those empirical patterns meant in practice.

### Ethical Considerations

Ethical approval for the original study was granted by the Faculty of Health, Medicine, and Life Sciences—Research Ethics Committee (FHML/HDT/2024.022). Before the interviews, all individuals were provided with detailed information regarding the study’s objectives and data management protocols. Written informed consent was obtained for both participation and audio recording. Additionally, all individuals specifically consented to the reuse of their data for related research. To protect participant privacy, all transcripts were pseudonymized and stripped of identifiers. No member checking was conducted, to avoid influencing participants’ perspectives in potential follow-up research.

## Results

### Overview

We selected the concepts of situated knowledge, standpoint theory, tinkering, situated value, and response-ability because they provide guidance for analyzing how knowledge, authority, and value are coproduced in sociotechnical systems, and illuminate the often-hidden social, institutional, and moral dimensions of AI adoption. This allowed us to interpret participants’ experiences not merely as practical challenges but as reflections of broader sociotechnical dynamics, including power relations, professional roles, and organizational constraints. Our analysis does not aim to list themes but to explore how clinicians and AI experts make sense of AI systems *in practice*. In each section, we first explain the STS concept and then follow with representative examples from our data. The aim of the quotations is not to provide statistical representation but to demonstrate how interpretations are grounded in participants’ accounts. The STS concepts did not, therefore, determine what was found; rather, they provided conceptual resources for interpreting patterns from the empirical material.

### Situated Knowledge

A central concern across the interviews was how much and what kinds of information clinicians need about AI-CDSS used in practice. This was expected, given that the interviews focused specifically on clinicians’ informational needs. However, the desired level of information varied across clinicians, depending on individual background and the specific clinical and technical context in which the AI system was being used. While some clinicians described a lack of access to useful information about the workings of the AI model or how it was developed and validated, others experienced the provided information as too technical and not grounded in clinical practice. Similarly, AI experts reported struggles in deciding what information to communicate to clinicians.

*I think clinicians should know all the shortcomings that are there, but you also do not want to overwhelm them with the information that they cannot use.*

[Expert 3]

Our data reveal that the information clinicians want to receive to *understand* AI systems is shaped by their professional roles, prior experience with AI, and the specific clinical contexts in which AI-CDSS are used.

*And I think that for us simple doctors, to put it that way, we are not going to be able to do without AI specialists if we want to work with this [AI model]. You will not be able to understand everything. You won’t have to understand everything either.*

[Clinician 2]

The notion of *situated knowledge* was initially introduced by Haraway [39], challenging the belief that there is one single universal truth. Instead, they put forward that all knowledge is partial (incomplete), situated (produced from a specific positioning in the world), and multiple (different perspectives can coexist). This means that knowledge does not exist in a value-free space but is shaped by the social and cultural structures it is embedded in. By *situating* knowledge, one aims to show from which perspectives and viewpoints knowledge comes. This creates possibilities for accountability, as understanding the origin and cultural context of a knowledge claim enables it to be held to account.

What counts as relevant information for one clinician is excessive or irrelevant to another. These differences show that there is no *universally fixed* informational package that enables clinicians to make sense of an AI system. This introduces tensions in epistemic coordination, where information is defined in different ways and only becomes meaningful in relation to the practices, responsibilities, and decisions of those engaging with it.

### Standpoint Theory

Within the interviews, clinicians emphasized that understanding how to interpret and act on the AI-CDSS’ output is even more important than grasping the inner workings of a model. The *usefulness* of an AI system’s recommendation depends on the clinician’s ability to integrate it meaningfully into their decision-making process. Clinicians stressed that they do not want to be handed an output, but rather to understand what to do with it. This meant needing a sense of the AI system’s limitations, its range of applicability, and how much weight to give it relative to their own judgment to be able to act on the output.

*That you have a good model that is also clinically relevant. And with all due respect, but an information specialist should not be able to judge that. He is not trained for that. And a clinician is not trained to develop that model, I think you have to do it together in the development phase.*

[Clinician 2]

Feminist standpoint theories, as originally coined by Harding [40] and Collins [41], build on the idea of situated knowledge by emphasizing the importance of making explicit where knowledge claims come from. They argue that knowledge originates from certain cultural and social positionings—or standpoints—and is therefore riddled with values and influenced by power relations. To avoid reinforcing epistemic inequalities (which are differences in whose knowledge and experiences are recognized, valued, and trusted), it is important to clarify the standpoint of knowledge claims. Standpoint theory emphasizes centering perspectives from less-heard or underrepresented groups, explicitly acknowledging context, and actively pursuing balance between standpoints.

Clinicians emphasized understanding how to interpret outputs and integrate them into decision-making, while AI experts focused on model performance metrics and hyperparameters. Information that is useful to AI experts is not always the same as information that is useful to clinicians.

*The more you go into that statistic, the more difficult it becomes of course, see, the doctors know what an AUC is and the higher the better. They also know that it is never 1. That 0.5 is random. But then it stops. That is that then. I think that majority even think that that applies to all the patients they then throw into that model that it is not even at subgroup level that some predict better, others don’t.*

[Expert 2]

### Tinkering

In the interviews, clinicians reported that initial engagement with AI-CDSS often happens through experimentation. In cases where structured onboarding or formal training was lacking, clinicians developed their own strategies to make sense of the AI system. This included entering different inputs into the system to observe how the output would change and was carried out both before using the AI in clinical practice at all—with fictional information entries—and with a real-world patient. For example, if an 80-year-old patient seems more vital than the average 80-year-old, one clinician stated that they would type an age of 70 years into the AI-CDSS to find out how a more vital patient would respond to the intended treatment. This was not a formal validation procedure, but a way for clinicians to learn how the system’s output changes across different situations and how these relate to their own clinical assessment.

*Age is just a number, isn't it? (...) There are people in their 60s who function at the level of a 70-plus or even an 80-something (...) You are actually more vital than your age suggests, so that’s all interpretation. And I think you should be able to leave that to a clinician.*

[Clinician 8]

AI experts also reported that clinicians wish to test how the AI system behaves with data collected within their own institution (local validation). This distinct mode of engagement allows clinicians to explore how the system responds to their specific data, workflows, and clinical routines.

*I think doctors are especially good at external validation and generalizability, but also yes. I actually think that they would also like to test it internally themselves, want to pilot.*

[Expert 5]

Knorr [42] introduces the practice of *tinkering* in the context of a natural science laboratory, where the researchers’ “projective openness and the material character of operation and outcome” (p. 368) are part of the research process. Similarly, Mol et al [43] define tinkering as “attentive experimentation” (p. 13), a practice of trying out and adapting. Through this practice, experimentation is connected to the processes of valuation. Tinkering can involve adapting, adjusting, and improvising, which results in a form of reasoning in which appropriateness can be constructed through practice. This means that processes of valuation are not predetermined or defined in advance, opening up possibilities for alternative forms and standpoints in knowledge creation.

These findings highlight different forms of experimentation, or tinkering, through which clinicians test the AI system’s behavior and draw conclusions about when and how to (mis)use it. Tinkering refers not simply to experimentation or trial-and-error, but to the practices and negotiations through which clinicians develop an understanding of when and how AI outputs become meaningful.

### Situated Value

Participants emphasized that for AI-CDSS to be meaningfully adopted in clinical practice, they must contribute something beyond what clinicians already know. Some participants argued that if the AI-CDSS merely replicates clinical reasoning using the same variables, without introducing new perspectives or insights, its perceived value is limited. In these cases, AI systems were seen as adding complexity to an already complicated system. On the other hand, participants argued that AI should not aim to replace human expertise but rather augment it.

*There are a lot of tools, but actually no real applied decision models that we can use in the clinic. (...) Certainly not as a substitute for a choice. So yes, you do have certain models that can give you a bit of direction, but not really to make a choice.*

[Clinician 5]

From the clinicians’ standpoint, an AI-CDSS must generate actionable insights to be considered useful within clinical practice. Furthermore, our data indicate that AI experts tend to prioritize performance metrics and are not necessarily aware of different forms of valuation, which is further reinforced by publishing and reporting standards.

*Some doctors say, if I have to look something up in another system, I'm not going to use it. Others say, well, if it does help me, then I will and it will cost me a little more. So, there is also different parties between different parties. So, you can never make something that is good for everyone. But you do want, yes, not that you lose everyone on some points. (...) In the end you have 3 men who all do not use it for other reasons, so you want to have them all mapped out. And some can be solved by doing technical things. And others you have to tackle by education or information. Whether convincing and motivating people is often another thing.*

[Expert 2]

Heuts and Mol [44] draw from the work of Boltanski and Thévenot [45] on economies of worth and introduce the notion of *situated value*. This idea highlights that the worth of something is not fixed, but dependent on the context in which it is encountered. In their study of what counts as a good tomato, Heuts and Mol [44] interviewed *tomato experts* in the Netherlands and found that the same tomato could be valued in very different ways depending on which qualities played a role in its use. These differences in valuing are not simply in conflict with each other but can exist alongside one another. This reveals that multiple notions of what counts as *good* or *desirable* can be true at the same time. Similar to situated knowledge [39], the concept of situated value suggests that, instead of one singular, universal measure, values can be plural, relational, and shaped by the practices and intentions of those involved.

Interviewed clinicians judged an AI system’s usefulness based on whether it added something meaningful to their own clinical practice. For some, value meant gaining new insights; for others, it meant reducing uncertainty in decision-making. These different valuations contrast with those of AI experts, among whom higher performance metrics were the priority in constructing a *good* system. Therefore, value is not an inherent property of the AI-CDSS itself but is shaped by context, use cases, and the professional priorities of those encountering it.

### Response-Ability

Clinicians often raised the concern that the AI-CDSS output might be incorrect. Health care professionals are held accountable for the consequences of clinical decisions, including those that involve or are influenced by AI. Most interviewed clinicians argued that they lack technical expertise or resources to independently assess the AI system, which meant they had to place trust in others, often in the developers of the AI system. In addition, trust is also delegated to institutional structures, such as expert review boards or internal regulatory bodies, responsible for evaluating and approving AI systems for use in clinical environments. The lack of clear legal rules, standard training, or strong organizational guidance limits clinicians’ capacity to act on AI outputs accordingly.

*(...) But the Medical Device Regulation looks very much at, yes, say privacy things and reliability of the tool, but there is actually no clinical validation step to it. There are no guidelines for that and it is often very rattling. So, I'm actually looking for clearer guidelines about yes, just like you have to do phase 1 2 3 studies, about medicines of what do you have to do now before you can really use something reliable in practice. (...)*

[Clinician 4]

However, even AI experts have stated that they sometimes struggle to fully understand the functioning of an AI system, especially when contextual knowledge is lacking or when the AI system itself is difficult to interpret.

*In the end, you have a responsibility as a doctor to do the right thing, but within the framework of using it well, so to speak. When you use a model, and it doesn't work, you can eventually say, yes, but I still used the model, so to speak, you know. If you do not get it, you cannot really make an informed decision if you do not understand the information.*

[Expert 5]

*Response-ability* can be understood as the “capacity to respond” [46]. Originally introduced by Haraway [47], the concept describes an approach to finding ways of responding to situations, which is particularly relevant in contexts where the consequences or implications of certain actions are not immediately obvious. Response-ability invites reflection and thinking beyond causal reactions to explore alternative forms of response. These alternative forms of engagement should acknowledge the complex and interconnected nature of engagement. Therefore, things gain their meaning through interactions with other human and nonhuman entities. From this perspective, response-ability is not about accounting for every possible consequence. Instead, it calls for paying attention to possibilities, harms, and outcomes that might have more significant implications than expected.

These concerns illustrate how response-ability plays out in practice. Responding here is not only about reacting to an error when it occurs but also about *being able* to respond when AI outputs are uncertain, contested, or difficult to interpret. These concerns also depend on whether institutions provide the conditions and support needed for clinicians to act on uncertainty.

## Discussion

### STS Concepts as Sensitizing Lenses

The findings of this study highlight that the adoption and meaningful use of AI-CDSS in practice are not determined only by technical performance but also by the interplay of social, organizational, and moral relations. Our results do not challenge the well-established observation that technology adoption is shaped by social and organizational context, as demonstrated in existing frameworks of HCI, implementation science, and technology adoption research. This study extends these traditions by examining how clinicians’ and AI experts’ engagement with AI systems is shaped by the situated character of knowledge, the standpoint from which they operate, the ways they experiment and adapt tools in practice, the values they assign to AI outputs, and the organizational structures that they believe enable or constrain their capacity to respond. These findings resonate with broader insights from STS and health care technology research and extend them by highlighting how AI systems intersect with professional roles, institutional norms, and experiential expertise. Our contribution is not to claim that the general observation that technologies are shaped by their contexts is novel. Rather, this study applies feminist and constructivist STS concepts as sensitizing lenses to a specific contemporary case: the implementation of AI-CDSS, where questions of algorithmic authority, responsibility, and trust introduce particular challenges beyond conventional technology adoption.

### Context Matters

Participants described different informational needs in relation to their professional role, experience with AI, and clinical context. We interpret this variation through the concept of situated knowledge [39]. In practice, this could mean that information about the AI-CDSS needs to be specifically tailored to relevant stakeholders. Different users may benefit from different formats or levels of explanation depending on their role, expertise, and the decisions they need to make. Moreover, accountability emerges not simply as a property of the system, but as an effect of how information is articulated. This includes, for example, information about how data for the model were collected, as well as reflections on the structure and presentation of information to clinicians.

The situated character of how clinicians make sense of AI aligns with ethnographic and STS-informed studies on CDSS and medical technologies, which demonstrate that technologies are implemented differently across contexts and that adoption depends on the interplay between technology, organizational routines, and social interactions [48-53]. The variability we observed confirms that successful AI implementation requires attention to local practices, material infrastructure, and workflow integration.

### Multistakeholder Perspectives

Clinicians and AI experts emphasized different aspects of AI-CDSS when describing what they needed to understand to evaluate the model. We interpret these differences through standpoint theory [40,41]. Standpoint theory provides a framework for interpreting these differences as situated forms of knowing, rather than simply as differences in information preferences. AI experts have a different *standpoint* due to their professional roles and likely require different information from clinicians concerning AI-CDSS, such as performance metrics and hyperparameters. From the clinicians’ standpoint, a recommendation is only useful if it can be applied in practice, which highlights that integration extends to material processes, such as the availability of treatments, resources, and workflows in the clinical setting.

This echoes Mørk et al [54], who show that power relations within and across communities of practice shape whose knowledge is recognized and valued. Similarly, Breuer et al [55] demonstrate that engineers’ imaginaries influence health care technology design and engagement. These insights suggest that centering multiple standpoints is crucial to creating AI systems that are both usable and socially legitimate. Making these standpoints explicit and reflecting on them can help ensure that AI-CDSS are designed with the needs of their end users in mind to avoid overlooking expertise that comes from working directly with patients. Without this, they risk missing the realities of clinical care, rendering them not applicable in daily practice.

### Space for Experimentation

Participants’ descriptions of testing, interpreting, adapting, and locally validating AI-CDSS point to forms of work that extend beyond formal training and evaluation activities. Interpreted through the lens of tinkering, these activities make visible the often-unacknowledged work involved in making AI systems workable in practice [43]. Rather than relying on technical explanations, clinicians developed what might be described as lay expertise [56,57] based on their direct experience with the system’s output. This perspective draws attention to how practical knowledge about AI-CDSS can develop through experimentation and adjustment in use. This kind of knowledge is situated in clinical practice and grounded in relevance to specific cases, rather than in the inner workings or technical background of the AI system. The understanding that develops through experimentation may shape whether and how clinicians choose to rely on the AI system in practice.

The practice of tinkering resonates with prior research emphasizing the importance of incremental adaptation and situated experimentation in technology adoption [43,48,58]. Tinkering shows that knowledge about AI is coproduced through use, rather than passively acquired from technical documentation. This emphasizes the importance of looking beyond formal training or documentation and of highlighting the ways in which knowledge about AI is produced through use by nonexpert users, as well as of providing technical infrastructures for experimental learning and collaboration [59]. By applying this concept, we highlight the invisible labor involved in making AI systems workable in clinical practice, including the often-unacknowledged effort clinicians invest in testing, interpreting, adapting, and integrating AI systems beyond formal training and evaluation settings [60,61].

### Diverging Valuations

Participants differed in what they considered valuable about AI-CDSS, which suggests that the value attributed to AI-CDSS was related to participants’ professional roles and the purposes for which they anticipated using or evaluating the technology. We interpret this through the notion of situated value [44], showing that the perceived usefulness of AI-CDSS varies among stakeholders. What counts as *good* or *useful* AI depends on the perspective from which it is evaluated. This does not mean that one notion of value is superior to another, but rather that multiple, sometimes divergent, notions of value can be true at the same time. The differences in participants’ accounts suggest that AI-CDSS development needs to accommodate multiple professional perspectives on what constitutes useful and meaningful AI. Involving clinicians throughout the entire life cycle of the AI-CDSS, from conception until deployment and even afterward, and accepting that different perspectives and values shape the development of AI systems in divergent ways would be one way of doing that.

This aligns with STS analyses that argue that technology’s value is relational and context-dependent [16,62]. Recognizing these divergent valuations highlights the need for continuous involvement of multiple stakeholders throughout the AI life cycle, from design to deployment, ensuring that tools align with real-world clinical priorities rather than abstract technical ideals.

### Importance of Structural and Institutional Factors

Participants described uncertainty about responsibility in situations where AI outputs could be incorrect, while also identifying gaps in training, organizational guidance, and institutional support. We interpret these accounts through response-ability, which draws attention to the conditions that shape actors’ capacity to respond to AI-supported decisions [47]. Response-ability helps us interpret these accounts as pointing to the distributed conditions under which responsibility can be enacted, and it captures clinicians’ reliance on institutional structures and expert oversight when interacting with AI-CDSS. This entails anticipating possible problems and knowing how to act when perceived uncertainty is high. Clinicians rely on others to assess AI-CDSS, but these groups might lack necessary context or technical understanding. This is further complicated by a lack of clear legal frameworks, standardized training, and organizational guidance, with clinicians forced to navigate complex forms of delegated trust without adequate resources or support throughout the AI life cycle. Participants’ accounts suggest that explainability, validation, or certification alone may not address all of the concerns they associated with responsibility and appropriate use, particularly where training, organizational guidance, or institutional support were perceived as limited.

Uncertainties about the ability to respond to an AI system from the clinicians’ perspectives parallel feminist STS critiques of algorithmic authority, which show that technical expertise alone cannot account for the social, ethical, and political conditions that enable safe AI use [16,63]. From this perspective, safe and fair use of AI depends not only on how accurate the model is and what model information is communicated to clinicians but also on structural and institutional arrangements, which are necessary for clinicians’ use (or rejection) of AI.

### Limitations

The results of this study should be interpreted in light of several limitations, some of which are inherent to the original research design. First, the use of convenience and snowball sampling in the original study likely introduced selection bias, as individuals who volunteer to participate in AI research are often more technologically engaged or positive about the topic than the general clinical population. Consequently, our findings on *situated value* and *tinkering* may primarily reflect the sense-making of lead users or enthusiasts, rather than capturing clinicians more distanced from digital transformation. The findings should not be interpreted as representative of all clinicians’ perspectives or as capturing the experiences of those with limited engagement with AI-CDSS. As this is an interpretive qualitative study, our aim is not statistical generalization but to examine how meanings, values, and responsibilities surrounding AI-CDSS are constructed within specific professional and institutional contexts.

Second, the fixed sequence in providing participants with the Model Cards, TRIPOD-AI, and Model Facts may have introduced order effects, such as primacy bias. The heterogeneity of the specialties among the participant group also means that while we could identify broad themes of *situated knowledge*, we lacked the disciplinary depth to map out specific medical specialties in detail. Future research should examine in more detail how clinical workflows, organizational contexts, and specialty-specific forms of expertise shape the interpretation and integration of AI systems.

Finally, the original study was grounded in a post-positivist research stance, which constrained the depth of theoretical engagement. In particular, the research question foregrounded informational needs, a framing that aligns with instrumental and problem-solving approaches but does not constitute a central object of inquiry within STS. Moreover, in the initial interview design, participants were presented with multiple examples of reporting standards and asked to evaluate them. This approach diverges from STS methodologies, which typically privilege processual, narrative, and practice-oriented accounts over evaluative or opinion-based questioning. Finally, the interview guide itself mirrored these limitations by not exploring dimensions such as power relations, epistemic assumptions, and institutional constraints more explicitly.

### Conclusions

This study suggests that the adoption and meaningful use of AI-CDSS cannot be understood solely in terms of technical performance or informational provision. Instead, it is shaped by the social, political, and moral characteristics of the world they are supposed to become part of. These characteristics, and the recognition of diverse clinical and organizational contexts, are central to understanding technology adoption. As health care environments continuously evolve, our study provides a snapshot of how clinicians and AI experts in the Dutch context interpret and engage with AI-CDSS, highlighting the context-dependent and negotiated nature of AI uptake. To this end, feminist and constructivist STS concepts—situated knowledge, standpoint theory, tinkering, situated value, and response-ability—can provide a lens to examine more closely these *hidden* factors that are often ignored or only briefly discussed.

This methodological shift changes what is analytically foregrounded in the same interview material. For example, the original study analyzed clinicians’ and AI experts’ statements primarily in terms of the information they considered necessary. This analysis interprets these same accounts as expressing different forms of situated knowledge and professional standpoints, and therefore different criteria for what makes information meaningful. Similarly, participants’ accounts of experimentation can be read not only as a means of developing trust but also as practices through which clinicians develop situated knowledge about when and how AI outputs become usable, including the often-unacknowledged labor involved in testing and integrating the system. The divergence between clinicians’ emphasis on actionable clinical augmentation and AI experts’ emphasis on performance metrics, validation, and reporting requirements further illustrates that the value of an AI-CDSS is not assessed according to a single criterion but is negotiated across different professional practices and priorities. Finally, participants’ accounts of responsibility reveal that the capacity to respond to uncertain or contested AI outputs depends not only on the properties of the system or the information provided but also on institutional and organizational conditions.

In practical terms, these findings suggest that developing and implementing AI-CDSS requires continuous engagement with end users, attention to local workflows and decision-making practices, and the creation of institutional structures that enable accountability. Understanding AI adoption involves not only identifying social, technical, and organizational factors that influence implementation but also examining how decisions about appropriate performance and use are made in practice. This raises questions such as who determines what counts as an AI-CDSS working well and what constitutes appropriate use in a particular clinical setting. Developing detailed implementation frameworks or policy recommendations would require additional research specifically focused on intervention design, organizational implementation processes, or regulatory evaluation, which was beyond the scope of this qualitative interpretive study. However, incorporating STS perspectives can guide developers, policymakers, and clinicians to design AI systems that are socially legitimate, ethically grounded, and that can be integrated into clinical practice in a meaningful manner. This approach underscores that responsible AI adoption in health care depends as much on understanding the social and institutional conditions of use as on technical performance or communication strategies.

## Supplementary material

10.2196/99117 Multimedia Appendix 1 Interview guide clinicians and interview guide AI experts.

10.2196/99117 Multimedia Appendix 2 Examples of Model Card, TRIPOD-AI, and Model facts.

10.2196/99117 Checklist 1 COREQ checklist.

## Abbreviations

AI-CDSS: AI-driven clinical decision support systems
CDS: Clinical Data Science
CDSS: clinical decision support system
COREQ: Consolidated Criteria for Reporting Qualitative Research
HCI: human-computer interaction
HES: Department of Health, Ethics and Society
PUS: public understanding of science
STS: science and technology studies
TRIPOD-AI: Transparent Reporting of a Multivariable Prediction Model for Individual Prognosis or Diagnosis—Artificial Intelligence
